# A comparative analysis of the health status of men aged 60–72 years and men aged 73+ years in Jamaica: Are there differences across municipalities?

**DOI:** 10.4102/phcfm.v2i1.139

**Published:** 2010-09-10

**Authors:** Paul A. Bourne

**Affiliations:** 1 Department of Community Health and Psychiatry, University of the West Indies, Jamaica

**Keywords:** health status, Jamaica, men aged 60+ years, men aged 73+ years, older men

## Abstract

**Background:**

Since 1990, the number of older men (60+ years) in Jamaica has increased to in excess of 100 000, while there are 30 000 men aged 73+ years. This is despite the fact that men have higher mortality and morbidity rates than women and seek medical treatment less frequently than women. There exists, however, a dearth in literature regarding this phenomenon and, therefore, this study has endeavoured to reduce this gap.

**Objectives:**

The aims of this study were to, (1) model the health status of men aged 60–72 years in Jamaica, (2) model the health status of men aged 73+ years in Jamaica and (3) examine the disparity in health status of the two groups in order to ascertain the factors that influence the good health status of elderly men.

**Method:**

A sample of 1432 men aged 60+ years were extracted from a survey of 25 018 Jamaicans. Secondly, a sub-sample of 633 men aged 73+ years was extracted from the 1432 men aged over 60. The data from which those samples were extracted is called the Jamaica survey of living conditions (JSLC). The JSLC began in 1988 from a model of the World Bank's Living conditions survey and is a nationally cross-sectional probability sample. The current study used descriptive statistics to provide background information on the sociodemographic characteristics of the sample and logistic regressions were utilised to examine the factors that predict good health of men aged 60–72 years and men aged 73+ years in Jamaica.

**Results:**

The average age of the sample of men aged 60+ years was 71.14 years (SD = 5.64 years) and 78.5 years for the sample of men aged 73+ years (SD = 7.97 years). Approximately 63% of men aged 60–72 years indicated that their health was good compared to 53.3% for men aged 73+ years. Rural men recorded the least health status across the age cohorts. With regard to predictors of good health, the same factors were found to determine approximately 27% of the variability in good health. Ownership of health insurance was found to be the most influential predictor of good health and positive affective psychological condition the least significant predictor of good health for men aged 73+ years and second to last of five factors for older men.

**Conclusion:**

The highest self-reported good health was indicated by men aged 60–72 years and men aged 73+ years who dwelled in towns other than Kingston, the capital. The least good health was experienced by rural men. For older men, health insurance coverage does not indicate preventative health, but preparation for curative ill-health.

## INTRODUCTION

From 1880 to 1882, life expectancy at birth for women in Jamaica was 39.8 years compared to 37.02 years for men (Table 1). In 2004, more than a century later, women were outliving men by 6 years (Table 1). In Jamaica, population ageing is a feminised phenomenon. This is typically the same around the world. From 1950 to 1955, world statistics showed that life expectancy at birth for women was 47.9 years, compared to 45.2 years for men, indicating that former sex was outliving the latter by 2.7 years.^1, 2^ The disparity in life expectancy at birth between the sex cohorts increased to 4.2 years between 2000 and 2005.^1^ According to Jamaica's *Demographic statistics* ^3^, 10.9% of females were aged 60+ years, compared to 10.3% of males. For the world, in 2000, 11.1% of the female population was older than 60 years, compared to 8.9% of men. Concomitantly, world statistics indicated that a woman who is 60 years old is likely to live for an additional 20.4 years, compared to 17 years for men.1 Life expectancy is one of the indicators of the health status of an individual or population, which implies that women are enjoying a better health status than men.

**TABLE 1:** Life expectancy of Jamaicans at birth by sex: 1880–2004

Period	Average expected years of life at birth	

	Men	Women
1880–1882	37.02	39.80
1890–1892	36.74	38.30
1910–1912	39.04	41.41
1920–1922	35.89	38.20
1945–1947	51.25	54.58
1950–1952	55.73	58.89
1959–1961	62.65	66.63
1969–1970	66.70	70.20
1979–1981	69.03	72.37
1989–1991	69.97	72.64
1999–2001	70.94	75.58
2002–2004	71.26	77.07

*Source*: Jamaican demographic statistics (1972–2006)

Courtenay^4^ noted, from research conducted by the Department of Health and Human Services and Centers for Disease Control, that from the 15 leading causes of death (except Alzheimer's disease), the death rates were higher for men and boys in all age cohorts, compared to women and girls. Embedded within this theorising are the differences in fatal diseases explained by gender constitution,^5^ which Courtenay^5^ contributed to behavioural practices of the sexes that cause men to die approximately 6 years earlier than women.^6^

Studies have shown, however, that women have a higher propensity to contract particular conditions such as depression, osteoporosis and osteoarthritis.^7, 8^ Herzog^8^ noted that ’… it appears that older women are more likely to be impaired by their health problems, while older men (aged 60+ years) are more likely to die from them.’ A study that was conducted by Schoen et al.^9^ on a group of adolescents, revealed a different finding from what was reported by the other studies.^7, 8^ They found that men were more likely than women to feel stressed, ‘overwhelmed’, or ‘depressed’; they attributed this to the limited nature of men's social networks.

Schoen et al.^9^ found that men in general tend to be more stressed and less healthy than women and further argued that men are more likely to use denial, distraction, alcoholism, and other social strategies to conceal their illness or disabilities.^10–13^ On the other hand, Herzog^8^, referring to studies from a number of experts, wrote that women had higher rates of depression than their male counterparts. Could suicide among the aged be the result of depression? This is likely to be underreported, because other illnesses are often present and given as cause of death. Hertzog^8^ noted that data on suicide and depression yielded different results and, therefore, suicide is not necessarily an indicator of depression.

Along with a longer life expectancy, particularly for women, the number of years spent being unhealthy are on the rise.^14^ In an attempt to calculate the ‘quality of lived years’, the World Health Organization (WHO)^14^ developed the disability adjusted life expectancy (DALE) scale in order to account for unhealthy years in relation to life expectancy. The DALE does not only use length of years to indicate health and well-being status of an individual or a nation, but also incorporates the number of years lived without disabilities. The institution found that these accounted for a 14% reduction in life expectancy for poorer countries and 9% for more developed nations.

Jamaica is a developing country, which means that, according to the DALE, both sexes are experiencing 14 years of unhealthy life expectancy. In spite of this, yearly on average (since 1990), there are 565 men who cross the threshold of the life expectancy in Jamaica (72.3 years at birth). This figure is included in the 1842 men who cross the 60-year-old threshold annually; 30 of whom (8%) are older than their life expectancy at birth. Men and women are living longer, but the former seek health care less frequently (Table 2). Table 2 shows that men reported less illness/injury than women, sought less medical care and spent more time in health care facilities, all of which accounts for the disparity in life expectancy between the sexes.

**TABLE 2:** The gender composition of those who reported illness and sought medical care in Jamaica (in % age): 1988–2007

Year	Total		Reported illness		Sought medical care		Mean days of illness	
			
	Sought medical care	Health insurance coverage	Men	Women	Men	Women	Men	Women
1988	NI	NI	NI	NI	NI	NI	NI	NI
1989	8.2	54.6	15.0	18.5	44.7	52.8	10.6	11.1
1990	9.0	38.6	16.3	20.3	37.9	39.2	10.2	10.2
1991	8.6	47.7	12.1	15.0	48.5	47.4	10.0	10.3
1992	9.0	50.9	9.9	11.3	49.0	52.5	10.7	10.9
1993	10.1	51.8	10.4	13.5	48.0	54.7	10.7	10.1
1994	8.8	51.4	11.6	14.3	49.0	53.4	10.3	10.4
1995	9.7	58.9	8.3	11.3	59.0	58.9	10.6	10.7
1996	9.8	54.9	9.7	11.8	50.5	58.5	10.0	11.0
1997	12.6	59.6	8.5	10.9	60.0	59.3	11.0	10.0
1998	12.1	60.8	7.4	10.1	57.8	62.8	11.0	11.0
1999	12.1	68.4	8.1	12.2	64.2	71.1	11.0	11.0
2000	14.0	60.7	12.4	16.8	57.4	63.2	9.0	9.0
2001	13.9	63.5	10.8	15.9	56.3	68.2	9.0	10.0
2002	13.5	64.1	10.4	14.6	62.1	65.3	10.0	10. 0
2003	NI	NI	NI	NI	NI	NI	NI	NI
2004	19.2	65.1	8.9	13.6	64.2	65.7	11.0	10.0
2005	NI	NI	NI	NI	NI	NI	NI	NI
2006	18.4	70.0	10.3	14.1	71.7	68.8	9.7	10.0
2007	62.8	21.2	13.1	17.8	66.0	68.1	10.6	9.3

*Source*: Jamaica survey of living conditions, various issues

NI, no information was available.

Irrespective of the self-reported health conditions given by men, they experience higher rates of morbidity and mortality than women in Jamaica.^15^ The Jamaican Ministry of Health's publication^15^ showed that, of the five leading causes of death – malignant neoplasms, cerebrovascular disease, heart disease, diabetes mellitus and homicide – men outnumbered women in three of them. The risk of developing malignant neoplasms is 39% higher for men than for women and, similarly, 71.2 per 100 000 men develop heart disease, compared to 66.1 per 100 000 women. On the other hand, the risk of cerebrovascular disease is 14% higher, and diabetes mellitus is 64% higher, for women than for men.^15^

In 2007, approximately 11% of men were older than 60 years (*N* = 132 931, Table 3). Using the results from Table 3 and Bourne's^16^ earlier study, it was calculated that 40.2% of elderly Jamaicans reported suffering from at least one dysfunction (*N* = 118 603), 13.1% of men reported ill-health (*N* = 173 135), 75.1% of elderly people who reported ill-health had recurring ill-health (*N* = 89 071) and 72% of the elderly who had self-rated ill-health sought medical care (*N* = 85 394). It can be extrapolated from the data that approximately 5% of the 13.1% of self-assessed health conditions are accounted for by elderly men. Furthermore, it can be extrapolated from these statistics that 3.8% of elderly men expressed having recurring ill-health. Has the rationale for not studying older men's ill health conditions been due to the fact that only 5% were subject to such conditions?

**TABLE 3:** Number of older men (aged 60+ years) in Jamaica and difference over each year: 1990–2007

Year	Men aged 60+ years		Men aged 73+ years	
	
	Quantity	Difference over previous year	Quantity	Difference over previous year
1990	101 603	–	31 336	–
1991	110 350	8747	32 441	1105
1992	111 742	1392	32 966	525
1993	113 116	1374	33 488	522
1994	114 706	1590	34 073	585
1995	116 263	1557	34 635	562
1996	117 600	1337	35 158	523
1997	118 721	1121	35 605	447
1998	119 751	1030	36 022	417
1999	121 001	1250	36 505	483
2000	122 297	1296	37 003	498
2001	123 478	1181	37 459	456
2002	124 728	1250	37 940	481
2003	126 370	1642	38 541	601
2004	128 031	1661	39 149	608
2005	129 683	1652	39 754	605
2006	131 250	1567	40 033	279
2007	132 931	1681	40 948	915

*Source:* Calculations for men aged 73+ years were done by the author and the figures were extracted from the Jamaican demographic statistics, 2007.

Many studies have been done on elderly Caribbean nationals and Jamaicans in particular.^16–33^ An extensive review of the literature, however, showed that none have specifically examined men's health, or those factors that influence the good health of older men (aged 60+ years) in Jamaica. In spite of pressure by the WHO and some scholars in a drive to examine the social determinants of health^33–38^ in the Caribbean and, in particular in Jamaica, no work has been done on this subject area. This study is innovative, as it seeks to investigate the social, psychological and environmental determinants of the health status of older men. Studies on older Caribbean nationals are not the same as an investigation of the health status of older men (aged 60+ years) in Jamaica. The aims of this study were to, (1) ascertain factors that influence good health status of elderly men (aged 60+ years) in Jamaica, (2) ascertain the determinants of good health status of older men, (3) determine the potency of each variable and (4) distinguish between determinants of the men.

## METHOD

### Theoretical framework

Many studies have employed multivariate analyses in the examination of health status.^16, 17, 26, 39–44^ The use of econometric analysis in the study of health was developed by Michael Grossman^44^. This approach simultaneously captures biomedical and non-biomedical variables, unlike the bivariate analysis that is only able to investigate two variables. Based on the WHO's definition,^45^ health is inclusive of biomedical, socio-economic and psychological factors. Health, therefore, is determined by many factors and the use of an econometric model makes it possible to identify these. A multivariate model has a fundamental advantage over bivariate relations, as health is a multidimensional phenomenon; this model is able to capture more variables and without excluding some variables that cannot be accommodated in a bivariate association.

The theoretical framework that underlines the present work was developed by Bourne^17^ and is a modification of the work of Grossman^44^ and Smith and Kingston^43^. Grossman was the first to establish an econometric model to evaluate the health status of people. The model encapsulates some variables that determine health status of people in the world and can be represented in the following equation:

$$H_{t}=f(H_{t-1},G_{o},B_{t},MC_{t},ED)$$

In [Eqn 1], *H*_*t*_ represents the current health in relation to time period *t*. The stock of health in the previous period is shown by (*H*_*t-1*_), health behaviours, such as smoking, excessive drinking and good personal health is represented by *B*_*t*_, with exercise included as a separate variable – *G*_*o*_. The use of medical care *MC_t_*, education of each family member (ED) and all sources of household income (including current income) are also included.

Grossman's model was further expanded upon by Smith and Kingston to include socio-economic variables, as seen in the following equation:

$$H_{t}=H^{*}(H_{t-1},P_{mc},P_{o},ED,E_{t},R_{t},A_{t},G_{o})$$

This second equation expresses current health status *H*_*t*_ as a function of stock of health (*H*_*t-1*_), price of medical care *P*_*mc*_, the price of other inputs *P*_*o*_, education of each family member (ED), all sources of household income (*E*_*t*_), family background or genetic endowments (*G_o_*), retirement related income (*R*_*t*_) and asset income (*A*_*t*_).

Given that particular conditions influence the elderly differently from other age cohorts, Bourne used an econometric analysis to build a model that captures variables that influence the subjective well-being of elderly Jamaicans. This is represented by the following equation:

Wi=f(InPmc,ED,Ai,En,G,MS,AR,P,N,InO,H,T,V)

In [Eqn 3], W *i* is the well-being of the Jamaican elderly and is a function of cost of medical health care (*Pmc*), the educational level of the individual (ED), their age (*Ai*, where *i* is the individual), their environment (*En*), the gender of the respondents (*G*), their marital status (*MS*), their area of residence (*AR*), positive affective conditions (*P*), negative affective conditions (*N*), the level of household crowding (i.e. average occupancy per room) (*O*), their home tenure (*H*), their status as property owners (*T*) and their experiences of crime and victimisation (*V*).

### Design

This research study used secondary data collected jointly by the Planning Institute of Jamaica (PIOJ) and the Statistical Institute of Jamaica (STATIN). For this paper, the sub-sample was 1423 elderly men (aged 60+ years). The mean age of the sub-sample was 71.14 years (SD = 7.97 years). Another sub-sample was extracted from the survey, which comprised 633 men aged 73+ years (i.e. men living beyond the life expectancy, which is 72 years in Jamaica). The two sub-samples were extracted from a larger, nationally prevalent study, conducted between June and October 2002, of some 25 018 respondents. Stratified random sampling techniques were employed to design the survey (i.e. the Jamaica survey of living conditions [JSLC]) and detailed self-administered questionnaires were used to collect the data from the respondents. The questionnaire was modelled from the World Bank's Living standards measurement study (LSMS) household survey. There were some modifications made to the LSMS as the JSLC is more focused on policy impacts. The questionnaire covered questions on sociodemographic, economic and wealth variables, crime and victimisation, social welfare, health status, health services, nutrition, housing and physical environment. Interviewers who collected the data were trained to address the questions and concerns of interviewees. Data were stored and retrieved in the SPSS program (SPSS Inc; Chicago, USA) and, for the present research, descriptive statistics were used to provide certain sociodemographic characteristics of the sub-sampled population.

Based on the principles of parsimony (i.e. all variables that should be included were included), the final model only constituted those variables that were statistically significant (i.e. *p* < 0.05). This was attained by the using the health literature and the variables that were included within the framework of the current data set.

Demographic characteristics were provided for the sample and the sub-sample of men aged 60+ years and 73+ years. Logistic regression was used to establish, (1) a model for good health status of elderly men in Jamaica, (2) Wald statistics to examine the contribution of each significant variable in the model and (3) the odds ratios interpreted to address the difference within each variable.

Multivariate analysis (logistic regression) was used because the researcher wanted to test a number of variables simultaneously, and the fact that the dependent variable was binary; the most fitting statistical technique was logistic regression. The model that was tested in this study is adapted from Bourne's model [Eqn 3], to represent the following:

$$W_{i}=f(Pmc,ED,Ai,En,MS,AR,P,N,O,H,V)$$

In [Eqn4], W *i* is well-being of the elderly men in Jamaica and is a function of the cost of medical health care (*Pmc*), the educational level of the individual, their age (*Ai*), where *i* is the individual), their environment (*En*), their marital status (*MS*), their area of residence (*AR*), positive affective conditions (*P*), negative affective conditions (*N*), the level household crowding (i.e. average occupancy per room) (*O*), their home tenure (*H*) and their experiences of crime and victimisation (*V*). Property ownership (*T*) was omitted, owing to the number of missing cases (in excess of 15%).

The results were presented using unstandardised coefficients, Wald statistics, odds ratio (OR) and confidence interval (95% CI). The predictive power of the model was tested using the Hosmer and Lemeshow test,^46^ to examine goodness of fit of the model. The correlation matrix was examined in order to ascertain if auto-correlation (or multi-collinearity) existed between variables. Based on Cohen and Holliday^47^, the correlation can be weak (0–0.39), moderate (0.4–0.69), or strong (0.7–1.0). This matrix was used to exclude (or allow) a variable in the model. Wald statistics were used to determine the magnitude (or contribution) of each statistically significant variable in comparison with the others, and the OR for the interpretation of each significant variable.

### Analysis

#### Key terms

**Health:** This is defined as the self-rated health status of an individual.

**Good health:** This variable is derived from a number of questions that enquired about particular health conditions. It is a binary variable where, 1 = no reported ill-health and 0 = reported at least one health condition.^39^

**Age:** This is the total number of years lived since birth, measured from one birthday to the next.

**Psychological condition:** This is the psychological state of an individual, subdivided into positive and negative affective psychological conditions.

**Positive affective psychological condition:** This denotes hopefulness, optimism and life satisfaction. For this study this variable was measured using a number of responses with regards to being hopeful and optimistic about the future and life in general.

**Negative affective psychological condition:** This represents the degree to which an individual experiences feelings of hopelessness, pessimism and fear. In this study these were measured from a number of responses of a person experiencing loss of a breadwinner and/or family member, loss of property, loss of income and failure to meet household and other obligations.

**Household crowding:** This indicates the average occupancy of persons per room, that is, the total number of individuals in a household divided by the number of rooms occupied by the household (excluding the kitchen and bathroom).

**Married:** This is a binary variable, where 1 = those who indicated they were married and 0 = otherwise.

**Poverty level:** This is also a binary variable, where 1 = those people who are in the two poor quintiles (i.e. poorest and poor) and 0 = otherwise (i.e. those wealthier individuals in quintiles 3–5).

**Crime index:** In this study, the crime index was calculated using the following equation:

$$\text{Crime index}=\sum k_{i}T_{j}$$

This equation represents the frequency with which an individual witnessed or experience a crime, where *i* denotes 0, 1 and 2 (0 indicates not witnessing or experiencing a crime, 1 indicates witnessing one or two crimes and 2 indicates seeing three or more crimes). The variable *T_j_* denotes the degree of the different typologies of crime witnessed or experienced by an individual, where *j* is define by the following scale: 1 = valuables stolen, 2 = attacked with or without a weapon, 3 = threatened with a gun and 4 = sexually assaulted or raped. The summation of the frequency of crime by the degree of the incident ranges from 0 to a maximum of 51.

**Area of residence:** This is the general geographic locale in which an individual resides, where

1 = Kingston metropolitan areas (which are all the areas that are 100% urban) and 0 = otherwise, and 1 = peri-urban areas (which are places that are not 100% urban) and 0 = otherwise. The reference group is from a rural area.

## RESULTS

### Demographic characteristics of sampled population

#### Men aged 60+ years

Of the population of 1432 elderly respondents, the mean age was 71.14 ± 7.97 years (Table 4). A substantial majority of the population was married (50%), owned their own homes (85%), resided in rural areas (68%) and reported good health (63%). The majority of men had, at most, primary level education (62%); however, 3% had attained tertiary level education and 98.6% reported that they were the head of their household. Per capita income was evenly distributed, with 23.8% being in the wealthiest quintile. Furthermore, crime seemed to have minimal effects on the respondents.

**TABLE 4:** Sociodemographic characteristics of sample (*N* = 1432) for Jamaican men aged 60+ years

Characteristic	Number of respondents	Percentage
**Good health**		
No	528	37.6
Yes	878	62.6
**Marital status**		
Married	703	50.2
Never married	425	30.3
Divorced	30	2.1
Separted	32	2.3
Widowed	211	15.1
**Retirement income**		
No	1320	93.0
Yes	99	7.0
**Health insurance**		
No	1212	87.1
Yes	180	12.9
**Per capita income quintile**		
1 = poorest	265	18.6
2	252	17.7
3	286	20.1
4	281	19.7
5 = wealthiest	339	23.8
**Home tenure**		
Squatted or rent free	139	9.8
Rented or leased	82	5.8
Owned	1201	84.5
**Area of residence**		
Rural area	968	68.0
Peri-urban area	286	20.1
Urban area	169	11.9
**Head of household**		
No	20	1.4
Yes	1402	98.6
**Educational level**		
Primary or below	843	62.3
Secondary	462	34.3
Tertiary	41	3.0

Age (mean ± SD) = 71.14 ± 7.97 years; Average consumption per person (mean ± SD) = $JM80 654.69 ± $JM75 029.21; Household crowding (mean ± SD) = 1.15 ± 0.89 Crime (mean ± SD) = 1.5 ± 7.0.

#### Men aged 73+ years

Of the population of 633 men aged 73+ years, the mean age was 78.5 ± 5.64 years (mode year = 77, median = 77 years). A substantial majority of this sample was married (48.8%, *n* = 302), 23.6% never married (*n* = 146) and 22.6% widowed (*n* = 140). Most owned their own homes (88%, *n* = 557). In terms of area of residence, 70.9% (*n* = 449) resided in rural areas, 19.3% (*n* = 122) in peri-urban areas; and 9.8% (*n* = 62) in urban areas. A little more than half (53.5%, *n* = 333) reported good health. The majority of these elderly men had, at most, primary level education (67.4%, *n* = 402), while only 2.7% had attained tertiary level education. Almost all of the men (98.4%) indicated that they were the head of their households.

Per capita income was evenly distributed, with marginally more individuals being in the wealthiest quintile (23.4%, *n* = 148) and the poorest quintile (21.6%, *n* = 137). The average consumption per person in the men aged 73+ years was $JM77 877.07 (SD = $JM72 014) – at the time of the study the exchange rate was $US1.00 = $JM50.97. In addition, crime seems to have a minimal affects on men aged 73+ years.

### Analysis of logistic regression on good health of men aged 60–72 years

Of the 16 predisposed variables that were used in the model (Table 5), five were statistically significant (*p* < 0.5). The five factors that determined good health of older men in Jamaica – age, secondary education, health insurance ownership, area of residence and positive affective psychological conditions – accounted for 27.4% of the model (chi-square test (19) = 289.45, *p* = 0.001, -2 log Likelihood = 1419.72). Of the five predictors of good health, three negatively influenced health. These were age, secondary level education and health insurance. The model had statistically significant predictor power (model χ^2^ = 289.45, *p* < 0.001, Homer and Lemeshow goodness of fit χ^2^ = 12.84, *p* = 0.117) and correctly classified 73% of the sample (correctly classified 93% of those who had good health and 40% of those who did not report poor health).

**TABLE 5:** Logistic regression: Variables predicting the good health of men aged 60–72 years and 73+ years in Jamaica

Variable	Aged 60–72 years†			Aged 73+ years‡		
	
	Odds ratio	95% CI		Odds ratio	95% CI	
	
		Lower	Upper		Lower	Upper
Age	0.949	0.933	0.965***	0.961	0.929	0.994*
Secondary level education	0.640	0.488	0.840**	0.541	0.357	0.820**
Tertiary level education	2.327	0.816	6.633	1.755	0.378	8.140
Primary level education and below§	1	–	–	1	–	–
Medical expenditure	1	1	1	–	–	–
Married	0.959	0.731	1.259	0.963	0.651	1.424
Poor	1.174	0.854	1.613	1.269	0.792	2.032
Head of household	1.129	0.113	11.321	0.871	0.583	1.301
Environment	0.929	0.706	1.222	0.061	0.029	0.129***
Health insurance	0.055	0.033	0.092***	2.044	1.230	3.396**
Peri-urban areas	1.505	1.062	2.134*	2.195	1.053	4.577*
Urban areas	1.605	1.021	2.523*	0.283	0.073	1.104
Rural areas§	1	–	–	–	–	–
House tenure: Rent	0.758	0.360	1.595	0.500	0.231	1.085
House tenure: Owned	0.911	0.577	1.438	0.718	0.487	1.060
House tenure: Squatted§	1	–	–	–	–	–
Social support	0.807	0.621	1.050	0.986	0.755	1.286
Crowding	1.056	0.896	1.243	0.975	0.939	1.012
Crime index	0.994	0.977	1.011	1	0.933	1.071
Negative affective	0.976	0.934	1.021	1.097	1.010	1.191*
Positive affective	1.098	1.041	1.159**	1	1	1
Consumption per person	1	1	1	1	1	1

† Chi-square (df = 19) = 289.45, *p* = 0.001; Nagelkerke R-square = 0.274; -2 log Likelihood = 1419.72; Hosmer and Lemeshow goodness of fit *χ*^2^ = 12.843, *p* = 0.724.

‡ Chi-square (df = 18) = 132.21, *p* = 0.001; Nagelkerke R-square = 0.277; -2 log Likelihood = 653.92; Hosmer and Lemeshow goodness of fit *χ*^2^ = 14.47, *p* = 0.700.

§ Reference group.

* *p* < 0.05

** *p* < 0.01

*** *p* < 0.001.

Of those variables that negatively determined good health, ownership of health insurance carried the most weight in determining good health (Wald statistic = 122.88, 95% CI = 0.03–0.09, *p* = 0.001), followed by age (Wald statistic = 39.2, 95% CI = 0.93–0.97, *p* = 0.001). Embedded in these findings was the revelation that an individual who possessed health insurance is 0.06 times (odd ratio) less likely to experience good health compared to someone who does not have the same. Similarly, as older men age, they are 0.95 (odds ratio) less likely to have good health compared to a younger aged man. In addition, those who had obtained a secondary education, in comparison to primary level education, were 0.64 times (odds ratio) less likely to report good health (95% CI = 0.49–0.84). Furthermore, there was no statistical difference between men who had, at most, primary level education, compared to with tertiary level education, suggesting that those with primary level education have better health.

With respect to factors that have a positive effect on health, positive affective psychological conditions (Wald statistic = 11.67, 95% CI = 1.04–1.16) accounted for more variability than area of residence. On examining positive affective psychological conditions, it was found that when an elderly man experienced a positively affective condition, he was 1.1 times more likely to report good health. Findings revealed that men who reside in rural areas suffer from diminished good health. This means that those in peri-urban areas are 1.5 times (odds ratio, 95% CI = 1.06–2.13) more likely to report good health compared to an elderly man who dwelled in rural Jamaica. The elderly men who resided in the Kingston metropolitan area were 1.6 times (odds ratio, 95% CI = 1.02–2.52) more likely to report good health compared to those in rural Jamaica.

### Analysis of logistic regression on good health of men aged 73+ years

What factors account for good health in the men aged 73+ years? Table 5 shows that, of the 15 predisposed factors that tested for the initial model (good health of men aged 73+ years), five explain the variability in good health. These determine 27.7% of the variability in good health (chi-square (18) = 132.21, *p* = 0.001, Nagelkerke *R*-square = 0.277, -2 log Likelihood = 653.92). These five variables are: age, secondary level education, ownership of health insurance, area of residence and positive affective psychological conditions. Three of the explanatory variables negatively contribute to good health (age, secondary level education and health), and two positively affect good health (area of residence and positive affective psychological conditions).

The model had statistically significant predictor power (model χ^2^ = 132.21, *p* < 0.001, Hosmer and Lemeshow goodness of fit χ^2^ = 14.474, *p* = 0.070) and correctly classified 71% of the sample (correctly classified 84.9% of those who had good health and 55.1% of those did not report poor health).

Ownership of health insurance carried the most weight in determining good health (Wald statistic = 53.6, 95% CI = 0.029–0.129), followed by secondary level education with reference to primary level education (Wald statistic = 8.38, 95% CI = 0.357–0.820), living in peri-urban areas (Wald Statistic = 7.609, 95% CI = 1.230–3.396) and the least, dwelling in Kingston metropolitan area (Wald statistic = 4.396, 95% CI = 1.053–4.577). Embedded in these findings are the realisations that, (1) good health of men aged 73+ years are eroded with years of life, (2) those with primary level education enjoy a better self-reported health than those with secondary and tertiary level education, (3) owning health insurance does not positively contribute to good health, it is only an indicator of those who are likely to have poorer health, (4) men aged 73+ years who dwell in peri-urban areas are more likely to enjoy greater self-reported good health, followed by those who reside in the Kingston metropolitan area and, lastly, by those in rural areas and (5) men aged 73+ years that are experiencing more positive affective psychological conditions are 1.1 times more likely to report good health.

## DISCUSSION

All of the reports by the United Nations^1, 2^ and the WHO^14^, coupled with those of the Jamaican Ministry of Health^15^ and the JSLC that have been published on population, ageing, health or gender issues, have shown that women outlive men. The disparity in the life expectancy rate between the sexes is 6 years in Jamaica and 8 years using data on the world. Living longer means having to defy the odds of mortality for more years. This occurrence is accounted for by healthy lifestyle practices, implying that unhealthy lifestyle practices lead to higher mortality and morbidity in men than women. In spite of these realities, there are men living beyond the life expectancy in their respective geopolitical areas of residence. In Jamaica, the life expectancy for men is 72.3 years. The term the researcher has coined that refers to men who are alive beyond the life expectancy of their nation is men aged 73+ years.

Over an 18-year period (ending 2007), there were 40 948 men aged 73+ years in Jamaica and the average yearly increase of men aged 73+ years the period was 565. This means that there are men living beyond the expected life expectancy, mortality and disease-causing mortality rates. In spite of the high mortality and morbidity of men, this study provides information on what constitutes good health for older men.

Literature has shown that the good health status of people lessens as they become older.^48–52^ This study concurs with this finding, with 63% of men aged 60–72 years reporting good health, compared to 53.5% of men aged 73+ years, indicating that health decreases with ageing. This point was further reinforced by the finding in which age was a factor of good health in each of the models. Age as a factor was ranked as the second most influential in determining ‘good’ health (or lack thereof) for men aged 60–72 years compared to being one of the four influential factors for men aged 73+ years. However, age is not the only factor that affects good health of elderly men.

Other studies have shown that education influences good health and that tertiary level education is positively associated with better health.^4, 16, 17, 24, 26, 33–39, 43, 44^ This study agrees that education influences good health and that there is no difference between the health status of elderly men with primary and tertiary level education. Another interesting finding to emerge from this study is the fact that older men with primary level education in Jamaica have better self-reported health than those with secondary education. This contravenes other research that have shown that better quality education determines higher quality of health, but this is not the case for men who are living beyond 59 years.

Poverty, overcrowding, consumption and marital status in other studieshave shown to influence good health^17, 19, 24, 26, 43^; however, this is not the case for elderly men in this study. The fact that living beyond a particular year of birth (60 years) means that the individual has surpassed the need for certain material possessions and appetite for some foods; therefore, having financial resources or not, does not influence current health status – this goes for consumption, as well as other aforementioned variables that are not statistically significant (*p* > 0.05). Studies done about the elderly have indicated that overcrowding, consumption and marital status determine well-being, but in this study it was found not to be the case. The current work has refined the factors which effect elderly men and established that they are somewhat different from those that influence the health and well-being of elderly Jamaicans.

Although poverty does not directly relate to good health of elderly Jamaicans, good health has been found to differ based on area of residence. In 1997, the prevalence of poverty in the country was 9.9% and, 10 years later (2007), it had increased to 19.9%.^53^ Despite this exponential increase in the prevalence of poverty, 71.3% of the stock of poverty is accounted for by rural areas. Based on the data for 2007, 46.6% of elderly Jamaicans dwelled in rural areas, 20.9% in peri-urban areas and 32.5% in the Kingston metropolitan area.^53^ Within the context of the current study, it was found that the state of health of elderly men in rural areas were worse than other areas of residence and that poverty indirectly influences health. Furthermore, the best health is likely to be experienced by other town dwellers, but not those who dwell in the Kingston metropolitan area (100% cities). The stock of poverty for elderly residents of urban areas was 2.2 times (19.9%) greater than that of the distribution of poverty in peri-urban areas (8.9%), indicating that poverty indirectly determines good health of aged residents.

It has well established that positive affective psychological conditions are correlated to health^17, 54–60^ and this was concurred by the current study. Lyubomirsky^54^ shows that happier people view life in a positive manner. This attitudinal state explains how decisions are influenced and moods experienced. With a positive attitude, a better quality of life is experienced, as the individual thinks, acts, builds and carries out their life's task with more self-assurance.^55^ The contrary is also true – a pessimistic individual is more likely to have a lower self-esteem, less self-fulfilment and less self-actualisation than someone who is optimistic. DeNeve and Cooper^56^ have found that happier people are more optimistic and positive in nature. Diener and Seligman^57^ point out that moods are not stationary, thus happy people can have negative moods, but they do not dwell on the negatives indefinitely. Harris and Lightsey^58^ have established that negative affective conditions such as guilt, fear, anger and disgust inversely affect subjective well-being – just as positive factors directly influence well-being.^59^ However, this was not the case for elderly men in Jamaica. The literature has shown that the elderly seek more health care than any other age cohort, so their psychological state is directly influenced by their physical condition. If an elderly individual does not perceive that they have control over an illness or disability, it may result in self-destructive behaviour,^60^ which will negatively influence well-being. McCarthy^60^ offers a further justification for the correlation between psychological state and subjective well-being, when he writes that diabetic patients are six to seven times more likely to suffer from psychiatric illnesses, anxiety and depression than non-diabetic patients. In the current work, it was found that aged men who are experiencing positive affective psychological conditions are 1.1 times more likely to report good health and that this variable minimally contributes to good health for elderly Jamaican men.

Ownership of health insurance coverage does not only indicate health-seeking behaviour, it also means that men who have surpassed 60 years purchased more health insurance if they believed that they were more likely to become ill. Hence, health insurance is not a preventative measure; instead it is a product that is more demanded by this cohort of men who are more likely to report ill-health. This finding denotes that men aged 60+ years who own this product are using it as a cost reduction mechanism because they are aware that, as a result of their ill-health, the frequency with which they will need to visit health facilities will increase.

## CONCLUSION

In summary, the good health of men aged 60+ years deteriorates as they become older. This study has shown that there is no difference between the factors that determine good health of men aged 60–72 years and men aged 73+ years. Good health is strongly influenced by ownership of health insurance coverage, but not by positive affective psychological conditions. Men aged 60–72 years and men aged 73+ years who resided in rural Jamaica reported the least good health and the greatest self-reported good health was experienced by those in peri-urban areas. This study is the first of its kind as no literature exists with which to conduct a comparative study. This limitation, however, does not hamper it from providing insight into the health status of men aged 60–72 years and the factors which predict good health for this group, as well as men aged 73+ years.
